# HVEM Signalling Promotes Colitis

**DOI:** 10.1371/journal.pone.0018495

**Published:** 2011-04-18

**Authors:** Corinne Schaer, Stefanie Hiltbrunner, Bettina Ernst, Christoph Mueller, Michael Kurrer, Manfred Kopf, Nicola L. Harris

**Affiliations:** 1 Molecular Biomedicine, Institute of Integrative Biology, Swiss Federal Institute of Technology, Zurich, Switzerland; 2 Institute of Pathology, University of Bern, Bern, Switzerland; 3 Institute of Pathology, Cantonal Hospital Aarau, Aarau, Switzerland; 4 Swiss Vaccine Research Institute and Global Health Institute, Ecole Polytechnique Fédérale, Lausanne, Switzerland; Centre d'Immunologie de Marseille-Luminy, CNRS-Inserm, France

## Abstract

**Background:**

Tumor necrosis factor super family (TNFSF) members regulate important processes involved in cell proliferation, survival and differentiation and are therefore crucial for the balance between homeostasis and inflammatory responses. Several members of the TNFSF are closely associated with inflammatory bowel disease (IBD). Thus, they represent interesting new targets for therapeutic treatment of IBD.

**Methodology/Principal Findings:**

We have used mice deficient in TNFSF member HVEM in experimental models of IBD to investigate its role in the disease process. Two models of IBD were employed: i) chemical-induced colitis primarily mediated by innate immune cells; and ii) colitis initiated by CD4^+^CD45RB^high^ T cells following their transfer into immuno-deficient RAG1^-/-^ hosts. In both models of disease the absence of HVEM resulted in a significant reduction in colitis and inflammatory cytokine production.

**Conclusions:**

These data show that HVEM stimulatory signals promote experimental colitis driven by innate or adaptive immune cells.

## Introduction

Members of the TNFSF play a central role in the regulation of immune responses by providing signals involved in differentiation, activation, survival and homeostasis of immune cells [1]. HVEM can promote T cell proliferation and IFNγ production [2], [3], and has been linked to IFNγ production by human mucosal T cells [4]. HVEM has a widespread expression, being present on most hematopoietic cells in addition to some stromal and epithelial cells [5], [6]. HVEM has multiple ligands, however LIGHT is thought to be the predominant ligand delivering stimulatory signals in vivo [7]. LIGHT is expressed by activated T cells, immature DCs and monocytes [3], and binds to both HVEM and lymphotoxin β receptor (LTβR) which is present on stromal cells and some hematopoietic cells including DCs and monocytes [7]. LIGHT has been shown to induce the maturation of DCs as well as NK cell proliferation [8], [9]. LIGHT-deficient mice exhibit defective T cell proliferation and activation in vitro [3], and fail to reject MHC-mismatched cardiac allografts coinciding with decreased intragraft expression of IFNγ [10]. However, LIGHT-deficient mice display normal immune responses following infection with Mycobacterium tuberculosis [11] or influenza A [12], suggesting that LIGHT may regulate some cellular responses whilst being superfluous for others.

LIGHT is contained within a region of the human chromosome 19p13.3 identified as a susceptibility locus for IBD [13], and LIGHT mRNA transcripts are over-expressed in inflamed biopsies from IBD patients [14]. In an experimental model of IBD, transgenic over-expression of LIGHT on T cells resulted in a lymphoid proliferative disorder, widespread autoimmune disease and development of severe intestinal inflammation [15]. Intestinal inflammation driven by transgenic over-expression of LIGHT was found to involve signalling to both HVEM expressed by T cells and LTβR expressed by stromal cells [15]. Collectively, these data implicate, but do not prove, a role for HVEM stimulatory interactions in promoting intestinal inflammation.

In the current study we subjected mice deficient for LIGHT or HVEM to Dextran sulfate sodium (DSS)-induced colitis and investigated the impact of gene deficiency on diarrhea, ulcerations and cellular infiltration of the colon. Additionally, we compared the ability of wildtype C57BL/6 or HVEM^-/-^ and LIGHT^-/-^ CD4^+^CD45RB^high^ T cells to mediate experimental colitis following their transfer into immuno-compromised RAG1^-/-^ hosts. Our data demonstrates that HVEM-mediated stimulatory signals are essential for promoting innate and adaptive immune cell activation, pro-inflammatory cytokine production and intestinal pathology.

## Materials and Methods

### Mice

C57BL/6 mice, HVEM^-/-^ [16] mice and LIGHT^-/-^ [10] mice were bred and maintained under specific pathogen-free (SPF) conditions at Bio-Support (Zürich, Switzerland). HVEM^-/-^ and LIGHT^-/-^ mice were backcrossed for 9 generations to C57BL/6 background. RAG1^-/-^ (C57BL/6) mice were purchased from the Institute for Laboratory Animal Science, University of Zürich. Congenic CD45.1-allelic C57BL/6 mice were purchased from Jackson Laboratory. All mice used in this study were 5–8 weeks old. Mice from different genotypes were housed within the same cage or bedding from the cages of male mice was mixed for at least 2–3 weeks prior to all the experiments.

### Ethics Statement

All animal procedures were approved by the local animal committee Kantonales Veterinäramt Zürich, protocol no. 3282, and performed in accordance with our institutional guidelines.

### DSS- induced experimental colitis

Acute colitis was induced in age-matched C57BL/6, HVEM^-/-^, LIGHT^-/-^ and RAG1^-/-^ mice, by oral administration of Dextran sulfate sodium (DSS) (MP Biomedicals) at a concentration of 5% (w/v) in drinking water for 4 days. Age-matched C57BL/6, HVEM^-/-^ and LIGHT^-/-^ mice receiving normal drinking water served as controls. Mice were evaluated daily for changes in body weight or the development of clinical symptoms. Six days after the induction of colitis mice were sacrificed by CO_2_ inhalation, the abdominal cavity was exposed and the entire colon was removed from the cecum to the anus. As the distal colon is the main site of inflammation in the DSS model [17], the distal colon was analyzed for inflammation by mRNA expression for inflammatory cytokines and by histology.

### Experimental colitis induced by CD4^+^ T cells

T cell-mediated colitis was induced by transferring 4×10^5^ CD4^+^CD25^-^CD45RB^high^ T cells into RAG1^-/-^ mice. Cells isolated from spleen cell preparations were labeled with anti-CD4 micro-beads and separated by positive selection on a magnetic column, according to the manufacturer's instructions (Miltenyi Biotech). Purified CD4^+^ T cells were then labeled with CD4, CD25 and CD45RB antibodies and sorted for CD4^+^CD25^-^CD45RB^high^ population on a FACS Vantage (BD Biosciences). The purity of FACS sorted CD4^+^CD25^-^CD45RB^high^ cells was routinely ≥98%. Mice were sacrificed at 14 days, 28 days, or 6–8 weeks post transfer. In preliminary experiments the colon was divided into 3 parts and each part analyzed for inflammation by mRNA expression for inflammatory cytokines and by histology. As the middle section was repeatedly the most inflamed section in all mice we only analyzed this portion in later experiments and data is shown only for this section.

### Assessment of the clinical activity score during DSS-induced colitis

Assessment of body weight, stool consistency and the presence of occult/gross blood by a guaiac test (Hemoccult Sensa; Beckman Coulter) were determined at the day of sacrifice for all mice. Colitis was quantified with a clinical score, as described by Cooper et al. [17], using the parameters of weight loss, stool consistency and fecal blood. Briefly, weight loss was considered as negligible (0 points), 1–5% (1 point), 5–10% (2 points), 10–15% (3 points) or ≥15% (necessitating sacrifice and given 4 points). Stool character was characterized as normal (0 points), soft with well-formed pellets (2 points), or diarrhea (4 points). For occult blood, scores were given as an absence of blood (0 points), a positive hemoccult score (2 points) or gross bleeding (4 points). The scores for each individual parameter were then added together to give a total score between 0 and 12.

### Histological assessment of colitis

Colonic specimens obtained as described were fixed in formalin for at least 24 hours, embedded into paraffin, and cut into 4–5 µm sections. Sections were then stained with hematoxylin and eosin (H&E) for blind microscopic assessment of mucosal lesions. Histological scoring for DSS colon sections was performed, with slight modifications, as previously described by Schenk et al. [18]. Briefly, for inflammation scores were given as rare inflammatory cells in the lamina propria (0 points), increased numbers of lymphocytes and granulocytes in the lamina propria (1 point), confluence of inflammatory cells extending into the submucosa (2 points), or transmural extension of the infiltrate (3 points). For crypt damage scores reflected intact crypts (0 points), loss of every third crypt (1 point), loss of two out of three crypts (2 points), complete crypt loss (3 points), or change of epithelial surface with epithelial erosion (4 points). For evaluation of the confluence of epithelial erosion scores reflected an absence of epithelial erosion (0 points), 1–2 foci of epithelial erosion (1 point), 3–4 foci (2 points), or confluent epithelial erosion (3 points). The scores for each individual parameter were then added together to give a total score between 0 and 10.

Histological scoring of colonic sections from the T cell transfer-induced colitis model was determined according to the following parameters. An estimated score was made based on first impression (score from 0–3), loss of goblet cells (score from 0–4), crypt abscesses (score from 0–3), mucosal thickness (score from 0–3), cellular infiltration (score from 0–3) and epithelial erosions (score from 0–2). The scores for each individual parameter were then added together to give a total score between 0 and 18.

### Cell isolation and Flow cytometry

MLN single cell suspensions were obtained by mechanical disruption through a 40 µM cell strainer (BD Biosciences). Colon LP lymphocytes were isolated as previously described [19]. Briefly, colon tissue was cut into 0.5 cm pieces and incubated at 37°C for 30 min. in PBS containing 0.5% BSA, 2% HEPES, 1% NaPyruvate and 10 mM EDTA to remove epithelial cells. The remaining tissue was further digested in complete IMDM medium containing 10% FCS and 1.5 mg Collagenase VIII (Sigma Aldrich) for 20 min. at 37°C and then smashed through a cell strainer. Cells were stained with surface antibodies diluted in PBS with 0.5% BSA (Sigma Aldrich). For intracellular staining cells were fixed in BD lysis buffer (BD Biosciences), permeabilized using 0.5% Saponin (Sigma Aldrich) in 0.5% BSA/PBS and stained with intracellular antibodies in 0.5% Saponin in 0.5% BSA/PBS. For the analysis of cytokine production by intracellular staining cells were first stimulated with PMA (Sigma-Aldrich) and ionomycin (Sigma-Aldrich) for 4 h at 37°C in IMDM medium plus 7% FCS. For the final two hours, Brefeldin A (10 µg/ml) was added to the cultures to retain cytokines in the cytoplasm. Stained cells were analyzed on FACS Calibur (BD Bioscience) or Cyan (Dako Cytomation) flow cytometers using FlowJo software (Tree Star). Fluorescently conjugated mAbs directed against CD4 (L3T4), CD25 (PC61), CD45RB (C363-16A), CD45.1 (A20), Ki-67 (MOPC-21), IL-17A (TC11), IFNγ (XMG1.2) and Foxp3 (FJK-16s) were purchased from eBiosciences.

### Detection of cytokine mRNA expression by quantitative RT-PCR

Total RNA was isolated from all colonic specimens obtained as described using TRI Reagent (Molecular Research Center, Inc.) and reverse transcribed using Superscript III RT kit (Invitrogen). Transcribed cDNA was used as a template for the PCR reaction. Real-time RT-PCR was performed using Brilliant SYBR Green (Stratagene) and an iCycler (Bio-Rad Laboratories). Expression was normalized according to expression of the housekeeping gene β-Actin. Sequences of primers used: β-Actin; 5′-CTT TTC ACG GTT GGC CTT AG-3′ and 5′-CCC TGA AGT ACC CCA TTG AAC-3′, CCL3; 5′-AGA TTC CAC GCC AAT TCA TC-3′ and 5′-CCC AGG TCT CTT TGG AGT CA-3′, CCL4; 5′-TTC TGT GCT CCA GGG TTC TC-3′ and 5′-AGC AAA GAC TGC TGG TCT CA-3′, CCL5; 5′- CAA TCT TGC AGT CGT GTT TG-3′ and 5′- AGA ATC AAG AAA CCC TCT ATC-3′, IL-6; 5′-TTC CAT CCA GTT GCC TTC TTG-3′ and 5′-TCA TTT CCA CGA TTT CCC AGA G-3′, IFNγ; 5′-GCT CTG AGA CAA TGA ACG CTA C-3′ and 5′-TTC TAG GCT TTC AAT GAC TGT GC-3′, TNFα; 5-GAA CTG GCA GAA GAG GCA CT-3′ and 5′-AGG GTC TGG GCC ATA GAA CT-3′, CXCL9, 5′-GCA AAA GTG AGC TCC AGA AGG-3′ and 5′-AGC TTC CCA GAT CAC AGA GG-3′.IL-6; 5′-TTC CAT CCA GTT GCC TTC TTG-3′ and 5′-TCA TTT CCA CGA TTT CCC AGA G-3′, IL-12p40; 5′-TAC AGT TCA GGC GCC GGA T-3′ and 5′-AGA GTT AAC CTG AGG TCC GCA-3′, TNFα; 5-GAA CTG GCA GAA GAG GCA CT-3′ and 5′-AGG GTC TGG GCC ATA GAA CT-3′, IL-21R; 5′-TCT GGA CCA TCA CCT GTG TC-3′and 5′-TTG TGG CCA GAC CTG TGT AG-3′, IL23R; 5′-GCC AAG AAG ACC ATT CCC GA-3′ and 5′-TCA GTG CTA CAA TCT TCT TCA GAG GAC A-3′ and IL-6R; 5′-AAG AGT GAC TTC CAG GTG CC-3′ and 5′-GGT ATC GAA GCT GGA ACT GC-3′ .

### Statistical Analysis

A two-tailed unpaired Student's t test with a confidence interval of 95% was performed on all data and are shown as p-values p<0.05 (*), p<0.005 (**), or p<0.0005 (***).

## Results

### HVEM is required for DSS-induced colitis

**Figure 1:**
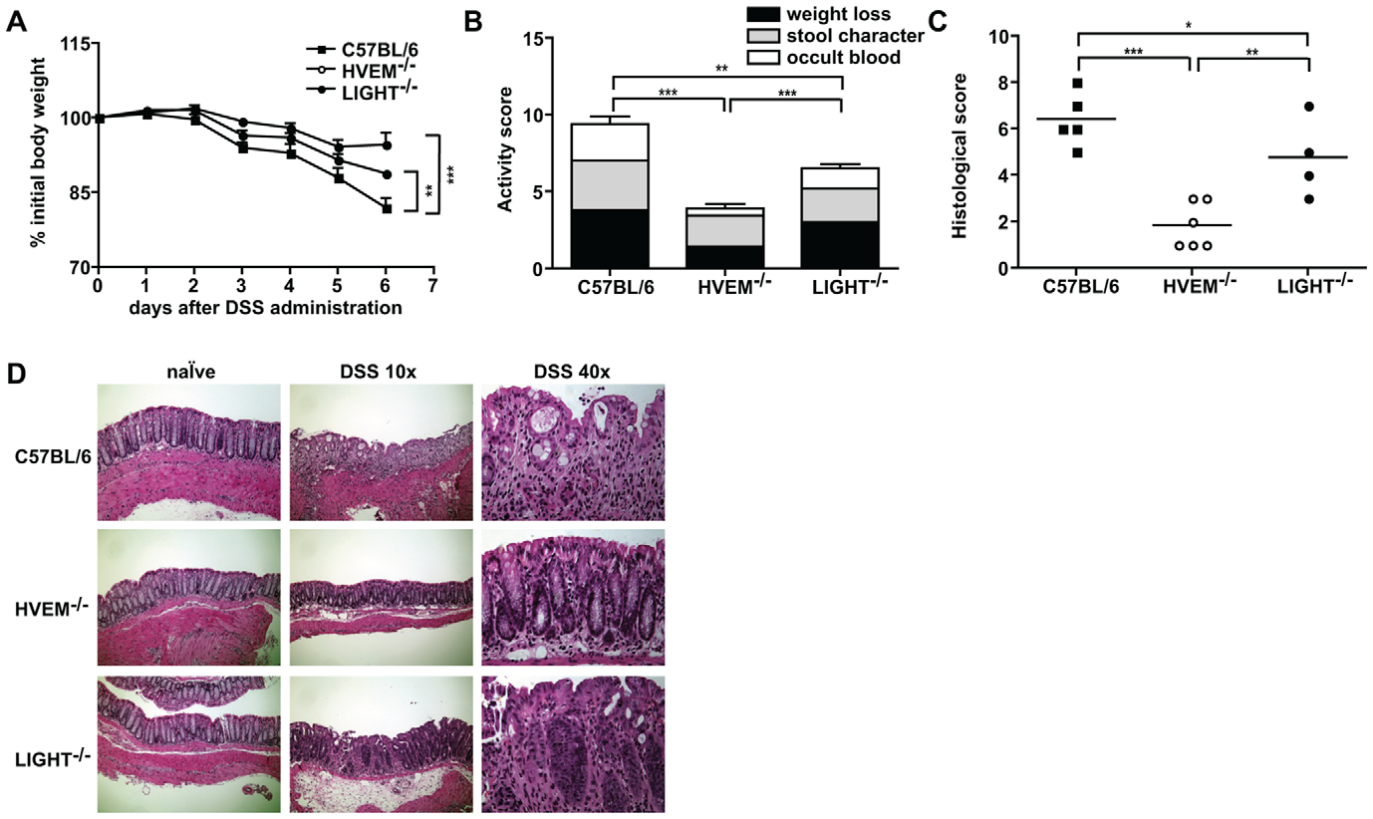
HVEM^-/-^ mice are resistant to DSS-induced colitis. C57BL/6, HVEM^-/-^ and LIGHT^-/-^ mice were given 5% DSS in the drinking water for 4 days, then returned to normal drinking water and sacrificed at day 6. (A) Body weight loss was monitored daily and is expressed as percentage change from initial body weight at day 0 for C57BL/6 (closed squares), HVEM^-/-^ (open circles) or LIGHT^-/-^ (closed circles). (B) Clinical activity scores were assessed at the time of sacrifice by a combination of total weight loss, stool character and occult blood. (C) Histological scores indicating immuno-pathology were calculated as described in the Materials and Methods and are shown for the distal part of the colon of individual mice for C57BL/6 (closed squares), HVEM^-/-^ (open circles) or LIGHT^-/-^ (closed circles). (D) Representative H&E staining of distal colon tissue sections from control and DSS-treated mice. Scale bars are 10× magnification  =  200 µm and 40× magnification  =  0.05 µm. Data in (A), (B) and (C) represent the mean ± SD of one experiment (n = 4-6 mice per group) out of three independent experiments. Statistically significant differences between groups were assessed by a two tailed Student's t test: ^*^p<0.05, ^**^p<0.005, ^***^p<0.0005.

To investigate the role of HVEM signaling during intestinal inflammation mediated by innate immune cells, we examined the response of HVEM^-/-^ mice to DSS-induced intestinal damage. In this model, administration of DSS in the drinking water results in weight loss, intestinal epithelial cell damage and immune-mediated colonic inflammation. As expected wild type C57BL/6 mice exhibited severe weight loss and intestinal inflammation following acute DSS administration (Figure 1A). In contrast HVEM^-/-^ mice exhibited significantly reduced weight loss and reduced rectal bleeding following DSS treatment (Figure 1A&B). In addition, HVEM^-/-^ mice showed attenuated intestinal immuno-pathology as determined by histological analysis of leukocyte infiltration, crypt destruction and epithelial erosion within the colon (Figure 1C&D). Importantly, the absence of inflammation in HVEM^-/-^ mice was not due to a delayed response, as mice did not exhibit weight loss even at late time points following DSS administration. Although DSS-induced intestinal inflammation can lead to the activation and recruitment of T cells, the disease is mediated largely by the activation of innate immune cells [20], [21]. Taken together, these data demonstrate that HVEM-mediated stimulatory signals are necessary to promote the activation of innate immune responses responsible for disease induction.

LIGHT is thought to represent a major HVEM stimulatory ligand. We therefore additionally investigated DSS-induced intestinal inflammation in LIGHT^-/-^ mice. LIGHT^-/-^ mice exhibited an intermediate phenotype showing less severe inflammation than C57BL/6 mice, but significantly more severe inflammation than that observed for HVEM^-/-^ mice (Figure 1A–D).

**Figure 2:**
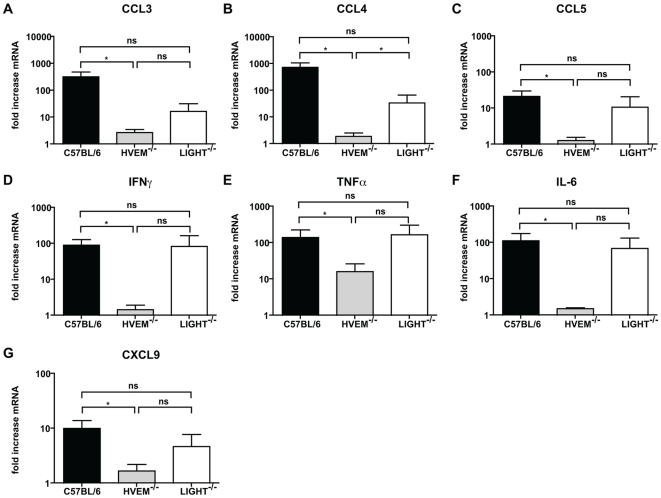
HVEM is required for DSS-induced pro-inflammatory cytokine and chemokine production. C57BL/6, HVEM^-/-^ and LIGHT^-/-^ mice were given 5% DSS in the drinking water for 4 days, then returned to normal drinking water. At day 6 mice were sacrificed and the colon removed for RNA isolation. (A) CCL3, (B) CCL4, (C) CCL5, (D) IFNγ, (E) TNFα, (F) IL-6 and (G) CXCL9 gene expression in the distal colon was analyzed by quantitative RT-PCR for C57BL/6 (black bar), HVEM^-/-^ (grey bar) and LIGHT^-/-^ (white bar) mice. For each individual sample, gene expression was normalized relative to β-Actin. Values represent fold increases in mRNA expression over the corresponding untreated controls. Means ± SD are shown for two independent experiments (n = 8–10 mice per group) out of three independent experiments. Statistically significant differences between groups were assessed by a two tailed Student's t test: ^*^p<0.05, ^**^p<0.005, ^***^p<0.0005.

To further assess the impact of HVEM on immunity during DSS-induced intestinal inflammation we analyzed the production of chemokines and pro-inflammatory cytokines within the intestine. DSS-induced production of CCL3 (MIP-1α), CCL4 (MIP-1β), CCL5 (RANTES), IFNγ, TNFα, IL-6 and CXCL9 was severely attenuated in the absence of HVEM (Figure 2A-G). These cytokines and chemokines are largely produced by local DC, monocytes and stromal cells, NK cells and/or T cells and their attenuation in the absence of HVEM indicates that HVEM is required for both innate and adaptive immune cell activation in this model. Chemokine and cytokine expression in LIGHT^-/-^ mice was highly variable but overall there was a non-significant decrease compared to C57BL/6 mice, and a non-significant increase as compared to HVEM^-/-^ mice (Figure 2A-G).

These data suggested that HVEM could signal directly to innate immune cells within the intestine to promote the production of pro-inflammatory cytokines and chemokines following DSS administration. The intermediate phenotype of LIGHT^-/-^ mice - in terms of chemokine and cytokine expression, weight loss and intestinal immuno-pathology - indicated that LIGHT only partially accounts for HVEM-mediated stimulatory signals in our model and raises the possibility that additional HVEM-stimulatory ligands contribute to intestinal inflammation in vivo.

### HVEM stimulatory signals regulate T cell-mediated colitis

**Figure 3:**
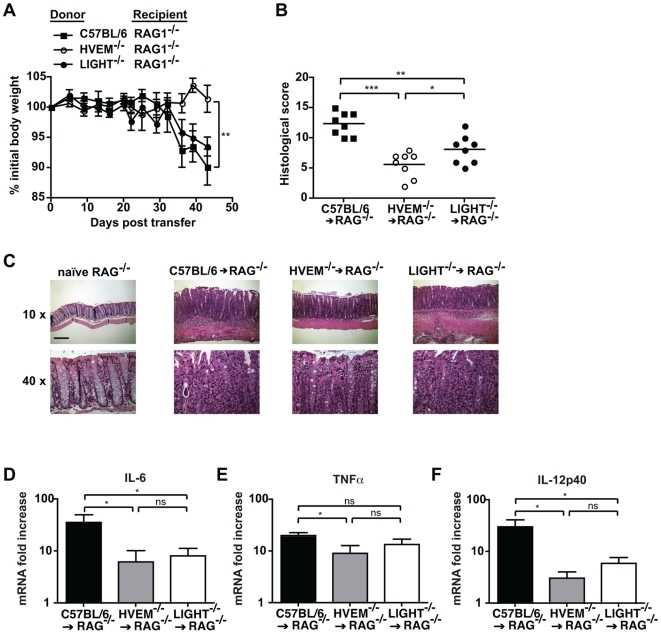
HVEM expression by CD4^+^ T cells is required for T cell-mediated colitis. 4×10^5^ CD4^+^CD25^-^CD45RB^high^ T cells from C57BL/6, HVEM^-/-^ or LIGHT^-/-^ mice were injected intravenously into RAG1^-/-^ hosts and recipients sacrificed 6-8 weeks later. (A) Body weight was monitored regularly and is expressed as percentage change from initial body weight at day 0 for C57BL/6 (closed squares), HVEM^-/-^ (open circles) or LIGHT^-/-^ (closed circles) T cells transferred into RAG1^-/-^ mice (n = 8 mice per group). (B) Histological scores indicating immuno-pathology were calculated as described in the Materials and Methods section and are shown for the middle part of the colon. Symbols represent individual RAG1^-/-^ animals receiving either C57BL/6 (closed squares), HVEM^-/-^ (open circles) or LIGHT^-/-^ (closed circles) CD4^+^CD25^-^CD45RB^high^ T cells (n = 8 mice per group). (C) Representative H&E staining of middle colon tissue-sections from control RAG1^-/-^ mice, and mice receiving either C57BL/6, HVEM^-/-^ or LIGHT^-/-^ CD4^+^CD25^-^CD45RB^high^ T cells. Scale bars are 10× magnification  =  200 µm and 40× magnification  =  0.05 µm. Colon mRNA expression of (D) IL-6, (E) TNFα and (F) IL-12p40 was determined in the distal colon part in RAG1^-/-^ hosts receiveing C57BL/6 (black bar), HVEM^-/-^ (grey bar) and LIGHT^-/-^ (white bar) CD4^+^CD25^-^CD45RB^high^ T cells. Data from (A), (B), (D), (E) and (F) represents means ± SD of two pooled experiments (n = 8 mice per group) out of four independent experiments. Statistically significant differences between groups were assessed by a two tailed Student's t test: ^*^p<0.05, ^**^p<0.005, ^***^p<0.0005.

To directly investigate the role of HVEM-mediated signaling to CD4^+^ T cells we used an experimental model whereby colitis is initiated by the transfer of CD4^+^CD45RB^high^ T cells into immuno-deficient hosts. CD4^+^CD45RB^high^ T cells were purified from the spleens of HVEM^-/-^ or wildtype C57BL/6 mice and 4×10^5^ cells injected intravenously into age and sex-matched RAG1^-/-^ recipients. RAG1^-/-^ recipients receiving CD4^+^CD45RB^high^ T cells from C57BL/6 mice exhibited weight loss and intestinal inflammation commencing between day 30 and 40 after transfer (Figure 3A–C). In contrast recipients of HVEM^-/-^ CD4^+^CD45RB^high^ T cells exhibited a clear resistance to weight loss (Figure 3A). Analysis of histopathology showed clear intestinal pathology in recipients of HVEM^-/-^ CD4^+^CD45RB^high^ T cells, however this was significantly reduced compared to RAG1^-/-^ mice receiving C57BL/6 CD4^+^CD45RB^high^ T cells (Figure 3B&C). The presence of some degree of intestinal inflammation in recipients of HVEM^-/-^ CD4^+^CD45RB^high^ T cells did not correlate with the absence of weight loss. However this was not unexpected as it is well described in this model that weight loss alone is not a reliable measure of intestinal inflammation [22].

Since T cells are known to be a major source of LIGHT [23], we additionally investigated the role of T cell-expressed LIGHT in promoting intestinal inflammation. Transfer of CD4^+^CD45RB^high^ T cells from LIGHT^-/-^ mice into RAG1^-/-^ recipients did not impact on weight loss (Figure 3A) but did reduce intestinal inflammation (Figure 3B&C). Attenuated intestinal inflammation observed following transfer of LIGHT^-/-^ T cells was significant when compared to mice receiving C57BL/6 T cells, but was not as dramatic as that observed for mice receiving HVEM^-/-^ T cells, indicating that other sources of LIGHT, or additional HVEM stimulatory ligands must exist.

To validate the reduced intestinal inflammation observed in mice receiving HVEM^-/-^ or LIGHT^-/-^ CD4^+^CD45RB^high^ T cells we additionally investigated the production of pro-inflammatory cytokines within the colon. All mice exhibited increased cytokine production as compared to naïve RAG1^-/-^ controls (Figure 3D–F). However, mice receiving HVEM^-/-^ T cells exhibited reduced levels of IL-6, TNFα and IL-12p40 as compared to recipients that received C57BL/6 naïve T cells, in the colon (Figure 3D, E&F). Mice receiving LIGHT^-/-^ T cells exhibited an intermediate phenotype compared to both C57BL/6 and HVEM^-/-^ CD4^+^CD45RB^high^ T cell-transferred mice, with significantly reduced IL-6 plus IL-12p40 in the colon (Figure 3D&F) but normal levels of TNFα (Figure 3E). Overall these data correlate well with the relative degrees of intestinal immuno-pathology observed in the same animals (Figure 3A–C) and reinforce our observations that HVEM stimulatory signals to T cells promote development of intestinal inflammation.

**Figure 4:**
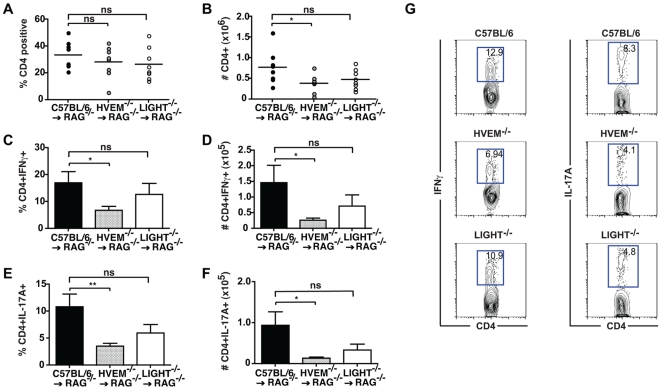
HVEM expression is required for the expansion and differentiation of CD4^**+**^ T cells during intestinal inflammation. CD4^+^CD25^-^CD45RB^high^ T cells (4×10^5^) from C57BL/6, HVEM^-/-^ or LIGHT^-/-^ mice were injected intravenously into RAG1^-/-^ hosts, recipients sacrificed 6-8 weeks later and total MLN lymphocytes isolated. Frequency of (A) CD4^+^, (C) CD4^+^IFNγ^+^ and (E) CD4^+^IL-17A^+^ T cells in C57BL/6 (closed circles and black bar), HVEM^-/-^ (grey circles and squattered bar) and LIGHT^-/-^ (open circles and white bar) transferred RAG1^-/-^ recipients were analyzed by flow cytometry using fluorescent marker-conjugated mAbs (n = 8 mice per group). Total numbers (indicated by # symbol) of (B) CD4^+^, (D) CD4^+^IFNγ^+^, (F) CD4^+^IL-17A^+^ T cells in the MLN of RAG1^-/-^ mice receiving either C57BL/6 (black bar), HVEM^-/-^ (striped bar) or LIGHT^-/-^ (white bar) CD4^+^CD25^-^CD45RB^high^ T cells were calculated (n = 8 mice per group). (G) Representative FACS profiles for IFNγ and IL-17A production by C57BL/6, HVEM^-/-^ or LIGHT^-/-^ CD4^+^ T cells isolated from the MLN of RAG1^-/-^ recipients. Symbols and bar graphs represent means (± SD) from two pooled experiments, and are representative of four independent experiments. Statistical analysis between groups was assessed by a two tailed Student's t test: ^*^p<0.05, ^**^p<0.005, ^***^p<0.0005.

We next investigated the impact of HVEM or LIGHT deficiency on the accumulation of T cells in the draining mesenteric lymph node (MLN) and their production of inflammatory cytokines. Mice receiving C57BL/6, HVEM^-/-^ or LIGHT^-/-^ CD4^+^CD45RB^high^ T cells had similar percentages of CD4^+^ T cells in the MLN (Figure 4A), however those mice receiving HVEM^-/-^ T cells had a decreased total number of CD4^+^ T cells present (Figure 4B). Both the percentage and total number of CD4^+^ T cells producing IFNγ or IL-17A was significantly decreased in mice receiving HVEM^-/-^ T cells as compared to mice receiving C57BL/6 T cells (Figure 4C–G). Selective deficiency of LIGHT on CD4^+^ T cells did not impact significantly on the total number of CD4^+^ T cells present in the MLN (Figure 4B), or on the percentage of CD4^+^ T cells producing IFNγ (Figure 4C) or IL-17A (Figure 4E). Total numbers of IFNγ^+^ or IL-17A^+^ LIGHT^-/-^ T cells were routinely observed to be decreased but this did not reach statistical significance (Figure 4D&F). By contrast, no differences in CD4^+^ T cell number and cytokine production were seen in C57BL/6, HVEM^-/-^ or LIGHT^-/-^ CD4^+^ T cells obtained from the spleen of transferred RAG1^-/-^ recipient mice (data not shown). These findings indicated that HVEM expression by CD4^+^ T cells is required for their expansion, activation or survival within the draining lymph nodes and for their effector function within the intestine during experimental colitis. Our data also demonstrated that the required HVEM-stimulatory signals were derived, at least in part, by T cell-expressed LIGHT.

### Attenuated CD4^+^ T cell expansion and cytokine production in the absence of HVEM stimulatory signals cannot be overcome by inflammatory conditions

**Figure 5:**
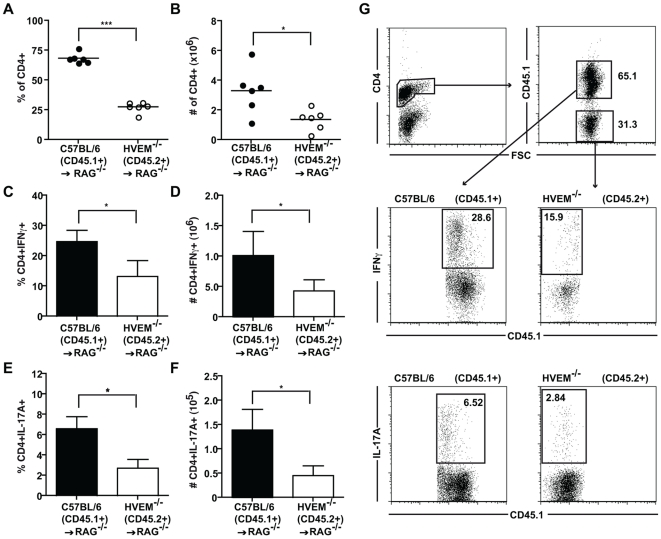
Attenuated HVEM^-/-^ CD4^**+**^ T cell expansion and cytokine production cannot be overcome by the presence of C57BL/6 CD4^**+**^ T cells. Purified 4×10^5^ CD4^+^CD25^-^CD45RB^hight^ T cells from congenic C57BL/6 (CD45.1^+^) and HVEM^-/-^ (CD45.1^-^) mice were injected together at a 1∶1 ratio (2×10^5^ cells each genotype) into RAG1^-/-^ recipients and mice were sacrificed 6-8 weeks later. Whole MLN cell suspensions from RAG1^-/-^ recipients were analyzed by flow cytometry and percentages of C57BL/6 (closed circles and black bar) and HVEM^-/-^ (open circles and white bar) (A) CD4^+^, (C) CD4^+^IFNγ^+^ and (E) CD4^+^IL-17A^+^ T cell lymphocytes assessed (n = 6 mice per group). Total numbers (indicated by # symbol) of C57BL/6 (closed circles and black bar) or HVEM^-/-^ (open circles and white bar) for (B) CD4^+^, (D) CD4^+^IFNγ^+^ and (F) CD4^+^IL-17A^+^ T cells were calculated (n = 6 mice per group). (G) Flow cytometry plots showing gating strategy to distinguish C57BL/6 (CD45.1^+^) from HVEM^-/-^ (CD45.1^-^) MLN CD4^+^ T cells recovered from RAG1^-/-^ recipients and representative plots of CD4^+^ T cell IFNγ and IL-17A cytokine staining. Symbols and bar graphs represent means ± SD from two pooled experiments and are representative of three independent experiments. Statistical analysis between groups were calculated using the two tailed Student's t test: ^*^p<0.05, ^**^p<0.005, ^***^p<0.0005.

We hypothesized that the decreased numbers of HVEM^-/-^ CD4^+^ T cells, and their inability to produce normal levels of inflammatory cytokines, may result from inadequate T cell-mediated DC activation and/or reduced production of pro-inflammatory cytokines in recipient mice. To address this we performed a co-transfer of equal numbers of HVEM^-/-^ and congenic C57BL/6 CD4^+^CD45RB^high^ T cells into RAG1^-/-^ recipients. Mice receiving both sets of T cells developed severe colitis similar to that observed in mice receiving C57BL/6 T cells alone (data not shown). However, both the percentage and total number of HVEM^-/-^ CD4^+^ T cells was markedly reduced in both the MLN (Figure 5A&B) and the spleen (Figure S1A&B) compared to their C57BL/6 counterparts. HVEM deficiency also resulted in reduced percentages and total numbers of IFNγ^+^ or IL-17A^+^ cells in the MLN (Figure 5C–G) and the spleen (Figure S1C–F). Thus, in contrast to our original hypothesis the co-transfer of C57BL/6 T cells allowed for the full development of inflammatory C57BL/6 T cells capable of initiating intestinal inflammation, but these cells acted in a competitive manner with HVEM^-/-^ T cells to further reduce their expansion.

### HVEM^-/-^ CD4^+^ T cells exhibit normal expansion at early time-points following their transfer into lymphopenic hosts

Our earlier findings indicated that HVEM deficiency on CD4^+^ T cells results in an inherent defect in the ability of these cells to expand and produce effector cytokines. We next set out to determine whether this defect occurred due to a failure to undergo homeostatic and/or spontaneous expansion following their transfer into lymphopenic hosts, or whether it was related to a defect in T cell activation and differentiation into effector cells. For this purpose a time-course experiment was performed whereby RAG1^-/-^ recipients receiving HVEM^-/-^ CD4^+^ T cells together with congenic wildtype C57BL/6 CD4^+^ T cells were sacrificed at day 14, 28 and 50 following transfer. The percentage and total numbers of CD4^+^ T cells was then determined and the fraction of CD4^+^ T cells expressing the proliferation marker Ki-67 examined.

**Figure 6:**
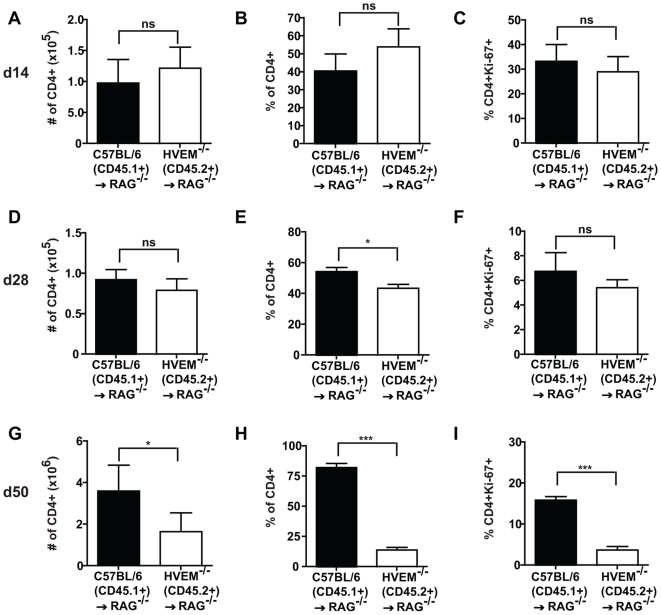
HVEM expression is required for the prolonged expansion of CD4^**+**^ T cells and differentiation of effector cells. 4×10^5^ CD4^+^CD25^-^CD45RB^hight^ T cells from congenic C57BL/6 (CD45.1^+^) and HVEM^-/-^ (CD45.1^-^) mice were injected together at a 1∶1 ratio (2×10^5^ cells each genotype) into RAG1^-/-^ recipients and mice were sacrificed at the indicated time points following transfer. MLN lymphocyte suspensions were counted and the total number (indicated by # symbol) of transferred C57BL/6 (CD45.1^+^, black bar) or HVEM^-/-^ (CD45.1^-^, white bar) CD4^+^ T cells determined. CD4^+^ T cell numbers at (A) day 14, (D) day 28 and (G) day 50 post transfer into RAG1^-/-^ recipient mice are shown. Frequencies of C57BL/6 (CD45.1^+^, black bar) or HVEM^-/-^ (CD45.1^-^, white bar) CD4^+^ T cells were assessed by flow cytometry at (B) day 14, (E) day 28 and (H) day 50 after injection into RAG1^-/-^ mice. Expression of the proliferation marker Ki-67 by C57BL/6 (CD45.1^+^, black bar) or HVEM^-/-^ (CD45.1^-^, white bar) CD4^+^ T cells. Data represent means ± SD of two pooled experiments (n = 6 mice per group) and are representative of three independent experiments. Statistically significant differences between groups (n = 6 mice per group) were assessed by a two tailed Student's t test: ^*^p<0.05, ^**^p<0.005, ^***^p<0.0005.

No differences in the percentage or total number of HVEM^-/-^ versus wildtype CD4^+^ T cells present in the draining MLN (Figure 6A&B) or colon (Figure S2A&B) were observed at day 14 following transfer. The fraction of CD4^+^ T cells expressing Ki-67 was also similar for HVEM^-/-^ and C57BL/6 cells (Figure 6C and Figure S2C). At day 28 following transfer the total numbers of HVEM^-/-^ CD4^+^ T cells, and the percentage of these cells expressing Ki-67 were not significantly different in the MLN (Figure 6D&F) or colon (Fig. S2D&F). However a significant reduction in the percentage of CD4^+^ HVEM^-/-^ T cells relative to C57BL/6 cells was noted for the MLN and colon at this time (Figure 6E and Figure S2E). In keeping with the data presented in Figure 5, both the percentage and total number of HVEM^-/-^ T cells was reduced compared to wildtype cells by day 50 post-transfer (Figure 6G&H and Figure S2G&H). This correlated with a decreased fraction of HVEM^-/-^ CD4^+^ T cells expressing the proliferation marker Ki-67 (Figure 6I and Figure S2I), and attenuated production of IFNγ and IL-17A (Figure 5). No significant production of IFNγ or IL-17A was noted for either C57BL/6 or HVEM^-/-^ T cells time-points earlier than day 50 (data not shown).

Taken together these data indicate that HVEM deficiency does not alter the ability of CD4^+^ T cells to undergo expansion, or to survive, during the first few weeks following their transfer into lymphopenic hosts. Instead defective T cell responses in the absence of HVEM appear to occur at approximately the same time as inflammation begins, indicating that they result from a reduced capacity to maintain expansion and to differentiate into effector cells in the presence of ongoing intestinal inflammation.

## Discussion

LIGHT-HVEM interactions have been previously implicated in IBD but the therapeutic potential of targeting this stimulatory pathway remains unclear. The aim of our study was to investigate how LIGHT- and HVEM-mediated stimulatory signals regulate intestinal immune responses during homeostasis or inflammation. For this purpose we used two mouse models of intestinal inflammation - where disease is driven primarily by innate cells or by CD4^+^ T cells and thus replicate distinct components of human IBD. These studies have allowed us to reveal that HVEM-mediated co-stimulatory signals to both innate immune cells and CD4^+^ T cells form an essential component of immune cell activation, proliferation, pro-inflammatory cytokine production and intestinal pathology.

Although we noted attenuated intestinal inflammation in the absence of HVEM, it is important to keep in mind that HVEM can also act as an inhibitory ligand. HVEM signaling to B and T lymphocyte attenuator (BTLA) [24] and CD160 [25] can result in an inhibition of T cell responses [24], [25], [26]. Indeed, inhibitory signals mediated by HVEM have been postulated to explain observations in HVEM^-/-^ mice of increased mortality during ConA-mediated autoimmune hepatitis [27], and increased susceptibility to MOG peptide-induced experimental autoimmune encephalomyelitis (EAE) [27]. In addition Steinberg et al. reported a critical role for HVEM expression by stromal cells in preventing intestinal inflammation following T cell transfer to immunodeficient hosts [28]. In keeping with our own observations, these authors saw a clear reduction in the histopathological score in the colon of RAG^-/-^ mice receiving HVEM or LIGHT deficient T cells [28]. However, they also reported an accelerated onset of intestinal inflammation following transfer of wildtype T cells into HVEM and RAG double deficient mice. This anti-inflammatory role of HVEM was determined to be mediated through T cell expressed BTLA [28]. Taken together with our own experiments these findings demonstrate that HVEM can mediate both pro- and anti-inflammatory signals during intestinal inflammation. We believe the most likely explanation of these collective data is that HVEM stimulatory signals are required to turn-on immune responses, whilst HVEM-mediated inhibitory signals function largely to switch-off immune responses. Support for this hypothesis comes from the finding that expression levels of the inhibitory ligands BTLA and CD160 are increased on T cells following their activation [24].

Although HVEM-mediated stimulatory signals were clearly required for the full activation of innate cells during DSS-induced colitis in our experiments, the exact identity of the stimulatory ligand(s) remains unclear. LIGHT is a known stimulatory ligand for HVEM, however LIGHT^-/-^ mice only exhibited a partial defect in intestinal inflammation indicating that an alternative ligand must exist. LTα3 is produced by activated T-, B- and NK-cells and has been reported to bind to HVEM in vitro [29]. Although not formally proven, it was postulated that LTα3 delivers stimulatory signals to HVEM based on the finding that both LIGHT and LTα3 bind to the CRD2 and CRD3 regions of HVEM [30]. Thus LTα3 and LIGHT together may deliver the stimulatory signals necessary to promote inflammation. Paradoxically, LTα-deficient mice are reported to exhibit increased disease severity following DSS administration suggestive of a regulatory role for LTα in this model [31]. However, it should be kept in mind that delineating a clear role for LTα3-HVEM interactions in vivo is difficult as LTα forms a heterodimer with LTβ which acts to stimulate stromal cell-expressed LTβ receptor (LTβR), an interaction that is essential for lymphoid organogenesis and organization (reviewed in [32]).

It is also possible that ligands other than LIGHT are responsible for HVEM mediated stimulatory signals during T cell mediated intestinal inflammation. In our experiments an absence of LIGHT on T cells did lead to a reduction in disease pathology but not to the same degree as T cell HVEM deficiency. Of course additional sources of LIGHT are likely to be present in the RAG1^-/-^ recipients in the form of resident innate cells (DCs, NK cells, monocytes), and these sources may account for the remaining disease observed in RAG1^-/-^ mice receiving LIGHT^-/-^ CD4^+^ T cells. Alternatively, CD4^+^ T cells may be able to receive HVEM stimulatory signals from other ligands such as LTα3. Lastly, Although BTLA-HVEM signaling is recognized to deliver inhibitory signals via HVEM, BTLA was recently shown to play a role in the accumulation T cells following T cell transfer into immunodeficient hosts [33]. This finding was attributed to an ability of BTLA to provide intrinsic survival signals to T cells following ligation of HVEM in a cis-complex and indicates than an absence of BTLA-HVEM signals may also play a role in the inability of HVEM^-/-^ T cells to promote intestinal inflammation [33].

## Supporting Information

Figure S1 **Reduced HVEM^-/-^ CD4^**+**^ T cell expansion and cytokine production in spleen cannot be overcome by the presence of C57BL/6 CD4^**+**^ T cells.** Purified 4×10^5^ CD4^+^CD25^-^CD45RB^high^ T cells from congenic C57BL/6 (CD45.1^+^) and HVEM^-/-^ (CD45.1^-^) mice were injected together at a 1∶1 ratio (2×10^5^ cells each genotype) into RAG1^-/-^ recipients and mice were sacrificed 6-8 weeks later. Whole spleen cell suspensions from RAG1^-/-^ recipients were analyzed by flow cytometry and percentages of C57BL/6 (closed circles and black bar) and HVEM^-/-^ (open circles and white bar) (A) CD4^+^, (C) CD4^+^IFNγ^+^ and (E) CD4^+^IL-17A^+^ T cell lymphocytes assessed (n = 6 mice per group). Total numbers (indicated by # symbol) of C57BL/6 (closed circles and black bar) or HVEM^-/-^ (open circles and white bar) for (B) CD4^+^, (D) CD4^+^IFNγ^+^ and (F) CD4^+^IL-17A^+^ T cells were calculated (n = 6 mice per group). Symbols and bar graphs represent means ± SD from one experiment. Statistical analysis between groups were calculated using the two tailed Student's t test: ^*^p<0.05, ^**^p<0.005, ^***^p<0.0005.
(TIF)

Figure S2 **HVEM expression is required for the expansion of CD4^**+**^ T cells in the colon during intestinal inflammation.** 4×10^5^ CD4^+^CD25^-^CD45RB^hight^ T cells from congenic C57BL/6 (CD45.1^+^) and HVEM^-/-^ (CD45.1^-^) mice were injected together at a 1∶1 ratio into RAG1^-/-^ recipients and mice sacrificed at the indicated time points after transfer. Colon lamina propria suspensions were counted and the total number (indicated by # symbol) of transferred C57BL/6 (CD45.1^+^, black bar) or HVEM^-/-^ (CD45.1^-^, white bar) CD4^+^ T cells calculated. CD4^+^ T cell number at (A) day 14, (D) day 28 and (G) day 50 post transfer into RAG1^-/-^ recipient mice. Frequencies of C57BL/6 (CD45.1^+^, black bar) or HVEM^-/-^ (CD45.1^-^, white bar) CD4^+^ T cells were assessed by flow cytometry at (B) day 14, (E) day 28 and (H) day 50 after injection into RAG1^-/-^ mice. Expression of the proliferation marker Ki-67 by C57BL/6 (CD45.1^+^, black bar) or HVEM^-/-^ (CD45.1^-^, white bar) CD4^+^ T cells was analyzed by flow cytometry at (C) day 14, (F) day 28 and (I) day 50 after injection. Data represent means ± SD of two pooled experiments (n = 6 mice per group) and are representative of three independent experiments. Statistically significant differences between groups (n = 6 mice per group) were assessed by a two tailed Student's t test: ^*^p<0.05, ^**^p<0.005, ^***^p<0.0005.
(TIF)
