# Supplementary figures and images for: HVEM Signalling Promotes Colitis
Source: PLoS One. 2011 Apr 18;6(4):e18495. doi: 10.1371/journal.pone.0018495 (PMC3078914; doi:10.1371/journal.pone.0018495)

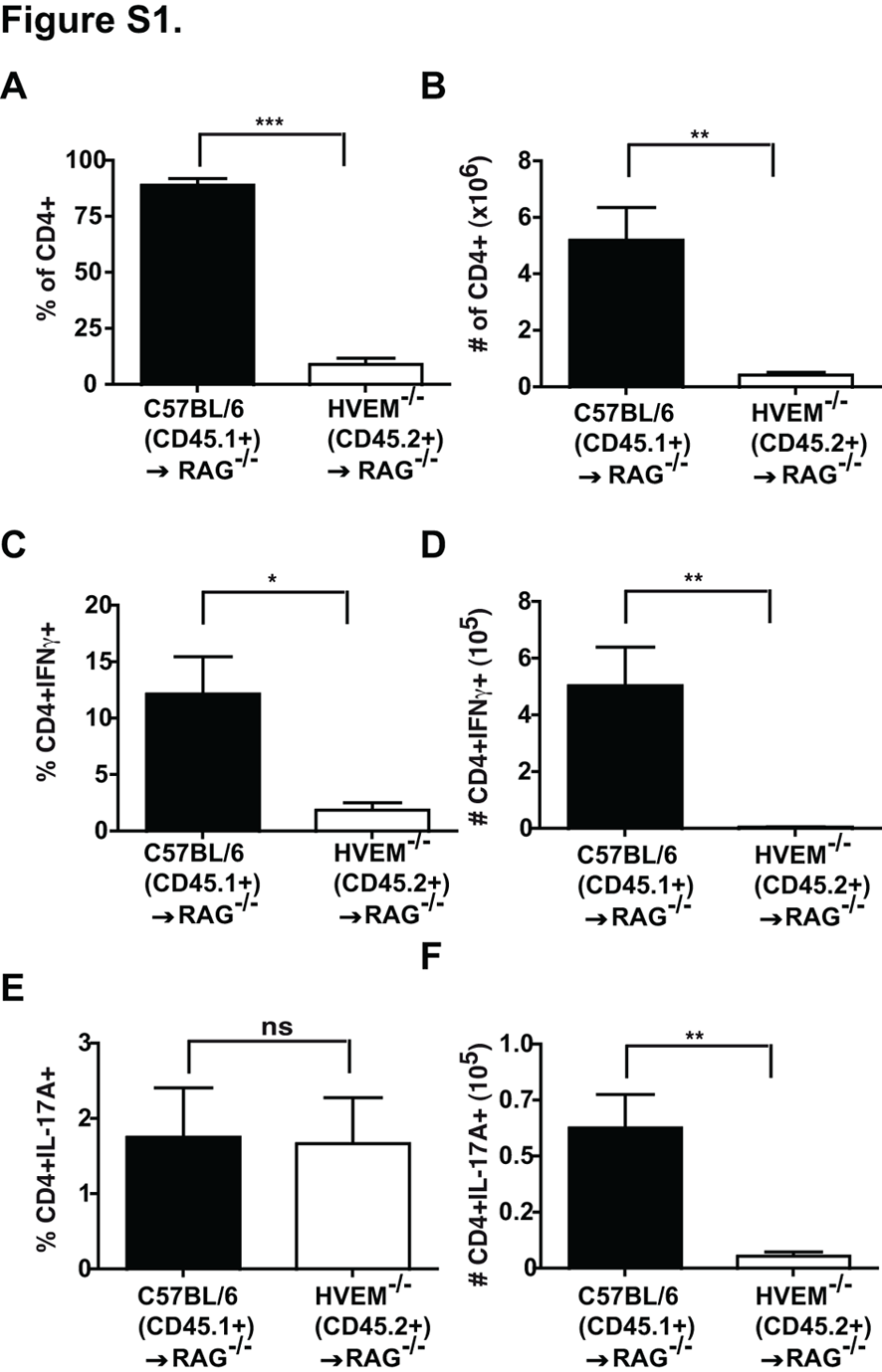

Supplement: Figure S1 — Reduced HVEM-/- CD4+ T cell expansion and cytokine production in spleen cannot be overcome by the presence of C57BL/6 CD4+ T cells. Purified 4×10^5^ CD4+CD25-CD45RBhigh T cells from congenic C57BL/6 (CD45.1+) and HVEM-/- (CD45.1-) mice were injected together at a 1∶1 ratio (2×10^5^ cells each genotype) into RAG1-/- recipients and mice were sacrificed 6-8 weeks later. Whole spleen cell suspensions from RAG1-/- recipients were analyzed by flow cytometry and percentages of C57BL/6 (closed circles and black bar) and HVEM-/- (open circles and white bar) (A) CD4+, (C) CD4+IFNγ+ and (E) CD4+IL-17A+ T cell lymphocytes assessed (n = 6 mice per group). Total numbers (indicated by # symbol) of C57BL/6 (closed circles and black bar) or HVEM-/- (open circles and white bar) for (B) CD4+, (D) CD4+IFNγ+ and (F) CD4+IL-17A+ T cells were calculated (n = 6 mice per group). Symbols and bar graphs represent means ± SD from one experiment. Statistical analysis between groups were calculated using the two tailed Student's t test: *p<0.05, **p<0.005, ***p<0.0005. (TIF) [file pone.0018495.s001.tif]

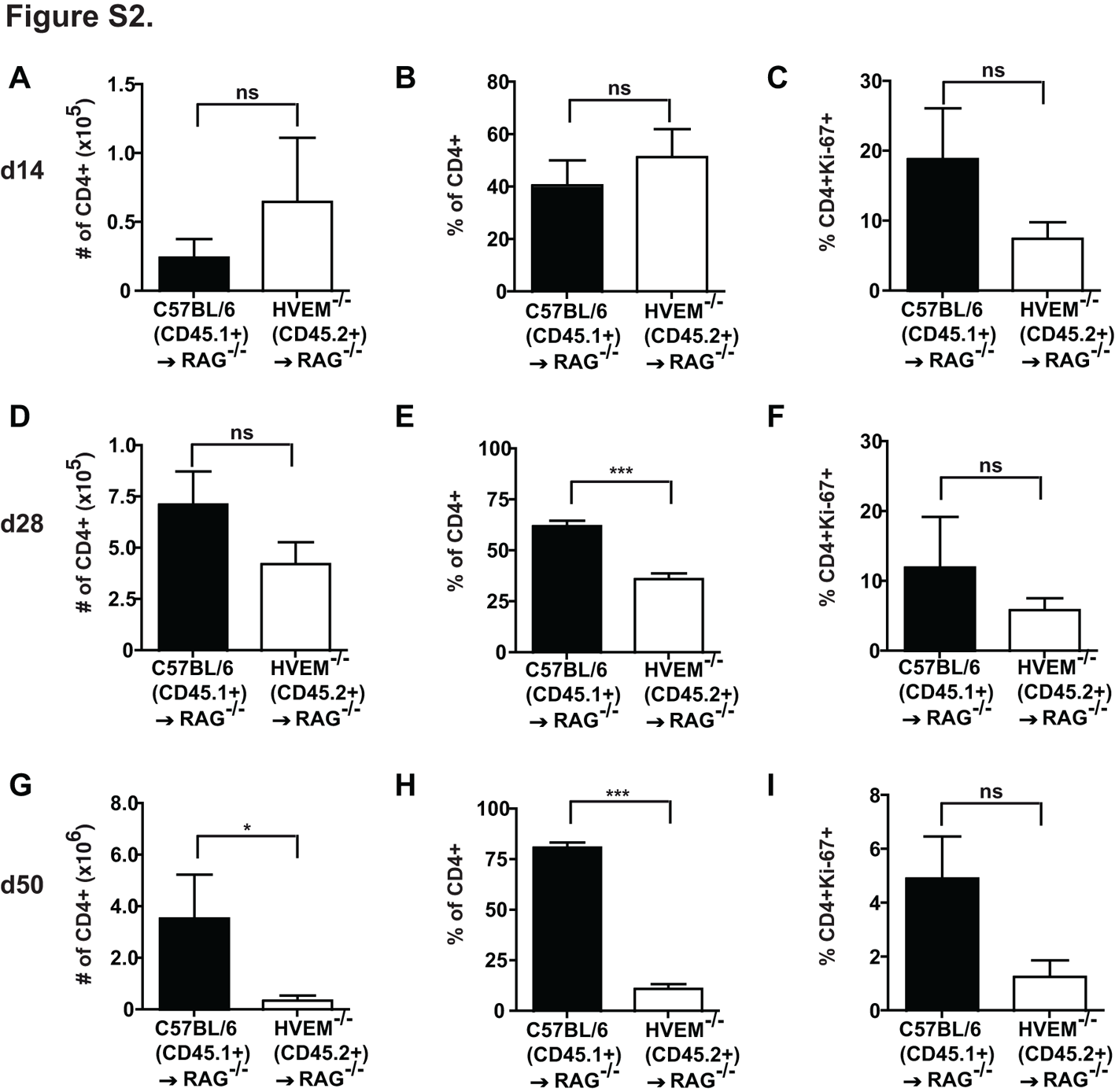

Supplement: Figure S2 — HVEM expression is required for the expansion of CD4+ T cells in the colon during intestinal inflammation. 4×10^5^ CD4+CD25-CD45RBhight T cells from congenic C57BL/6 (CD45.1+) and HVEM-/- (CD45.1-) mice were injected together at a 1∶1 ratio into RAG1-/- recipients and mice sacrificed at the indicated time points after transfer. Colon lamina propria suspensions were counted and the total number (indicated by # symbol) of transferred C57BL/6 (CD45.1+, black bar) or HVEM-/- (CD45.1-, white bar) CD4+ T cells calculated. CD4+ T cell number at (A) day 14, (D) day 28 and (G) day 50 post transfer into RAG1-/- recipient mice. Frequencies of C57BL/6 (CD45.1+, black bar) or HVEM-/- (CD45.1-, white bar) CD4+ T cells were assessed by flow cytometry at (B) day 14, (E) day 28 and (H) day 50 after injection into RAG1-/- mice. Expression of the proliferation marker Ki-67 by C57BL/6 (CD45.1+, black bar) or HVEM-/- (CD45.1-, white bar) CD4+ T cells was analyzed by flow cytometry at (C) day 14, (F) day 28 and (I) day 50 after injection. Data represent means ± SD of two pooled experiments (n = 6 mice per group) and are representative of three independent experiments. Statistically significant differences between groups (n = 6 mice per group) were assessed by a two tailed Student's t test: *p<0.05, **p<0.005, ***p<0.0005. (TIF) [file pone.0018495.s002.tif]
